# Dose-Related and Time-Dependent Development of Collagenase-Induced Tendinopathy in Rats

**DOI:** 10.1371/journal.pone.0161590

**Published:** 2016-08-22

**Authors:** Carlotta Perucca Orfei, Arianna B. Lovati, Marco Viganò, Deborah Stanco, Marta Bottagisio, Alessia Di Giancamillo, Stefania Setti, Laura de Girolamo

**Affiliations:** 1 Orthopaedic Biotechnology Laboratory, IRCCS Galeazzi Orthopaedic Institute, Milan, Italy; 2 Department of Drug Sciences, University of Pavia, Pavia, Italy; 3 Cell and Tissue Engineering Laboratory, IRCCS Galeazzi Orthopaedic Institute, Milan, Italy; 4 Department of Biotechnology and Biosciences, University of Milano-Bicocca, Milan, Italy; 5 Department of Veterinary Medicine (DiMeVet), University of Milan, Milan, Italy; 6 Department of Health, Animal Science and Food Safety, University of Milan, Milan, Italy; 7 IGEA SpA Clinical Biophysics, Carpi (MO), Italy; Queen Mary University of London, UNITED KINGDOM

## Abstract

Tendinopathy is a big burden in clinics and it represents 45% of musculoskeletal lesions. Despite the relevant social impact, both pathogenesis and development of the tendinopathy are still under-investigated, thus limiting the therapeutic advancement in this field. The purpose of this study was to evaluate the dose-dependent and time-related tissue-level changes occurring in a collagenase-induced tendinopathy in rat Achilles tendons, in order to establish a standardized model for future pre-clinical studies. With this purpose, 40 Sprague Dawley rats were randomly divided into two groups, treated by injecting collagenase type I within the Achilles tendon at 1 mg/mL (low dose) or 3 mg/mL (high dose). Tendon explants were histologically evaluated at 3, 7, 15, 30 and 45 days. Our results revealed that both the collagenase doses induced a disorganization of collagen fibers and increased the number of rounded resident cells. In particular, the high dose treatment determined a greater neovascularization and fatty degeneration with respect to the lower dose. These changes were found to be time-dependent and to resemble the features of human tendinopathy. Indeed, in our series, the acute phase occurred from day 3 to day 15, and then progressed towards the proliferative phase from day 30 to day 45 displaying a degenerative appearance associated with a very precocious and mild remodeling process. The model represents a good balance between similarity with histological features of human tendinopathy and feasibility, in terms of tendon size to create lesions and costs when compared to other animal models. Moreover, this model could contribute to improve the knowledge in this field, and it could be useful to properly design further pre-clinical studies to test innovative treatments for tendinopathy.

## Introduction

Tendinopathy is a big burden in clinics and it represents 45% of musculoskeletal lesions [1]. In particular, athletes and middle-aged people are frequently affected by tendinopathy of Achilles, patellar and supraspinatus tendons. The severity of tendon injuries ranges from transient pain and inflammation to chronic conditions leading to tears or total tendon ruptures [1, 2]. The poor healing capability of damaged tendons is related to their scarce blood supply and the compromised metabolic activity of resident cells [3] that determine an impaired tissue homeostasis [4]. The histopathological appearance of injured tendons shows collagen degeneration and fiber disorganization, increased vascularization and increased presence of resident cells, tissue metaplasia, and occasionally formation of fatty and bony deposits [5, 6]. However, despite its clinical significance, the pathogenesis and development of the tendinopathy are still under-investigated, thus limiting the therapeutic progress in this field. In fact, the current conservative treatments are still mainly symptomatic, whereas surgical approaches have a poor success rate and require a long recovery time [7].

In this contest, a better knowledge of tendinopathy progression throughout its phases could be reached through the development of an efficient *in vivo* model focused on the choice of the most effective dose of collagenase at an exact temporal window to induce the acute phase of the disease.

Despite an ideal animal model able to reproduce all aspects of human tendinopathy has not been identified so far, rat represents the most popular species to model the Achilles tendinopathy, thanks to its suitable size for surgical approaches and tissue retrieval, and its easy handling. Moreover, the rat model of Achilles tendinopathy has been extensively used in preclinical research, because of the similar conditions [8, 9] and the genetic homology to humans [10]. The most common techniques to develop rodent models of tendinopathy are based on mechanical overuse or chemical factors, such as collagenase, corticosteroids, cytokines (TGF-β1) and substance P [8, 9]. However, the mechanical overuse model is not completely accepted due to its scarce reproducibility and to the role of inflammation that does not equate to tendinopathy [8, 11]. Among the chemical-induced tendinopathy models, it has been shown that collagenase type I can provoke collagen fiber disruption and changes in biochemical and biomechanical features of the tendon, better resembling the main histopathological characteristics and functional impairments of human tendinopathy [8, 12, 13]. Thus, this injection model can represent a valid approach to induce and study the development of this pathology [14]. However, despite collagenase seems to be the most interesting agent to generate a consistent and reproducible model of tendinopathy, to date, standardized protocols have not been defined yet. Indeed, there is no agreement on which is the concentration, volume and site of injection, and time of occurrence of collagenase-induced lesions [13, 15–20].

The purpose of this study was to evaluate the cellular and tissue-level changes occurring in a collagenase-induced Achilles tendinopathy in rats at different time points by using two collagenase concentrations. In particular, we want to accurately investigate the development of the disease throughout its phases, in order to establish a standardized model for future pre-clinical studies that resembles as closely as possible the human pathology.

## Materials and Methods

### Ethics Statement

The Mario Negri Institute for Pharmacological Research (IRFMN) Animal Care and Use Committee (IACUC) approved the study (Permit N. 06/2014-PR). The IRFMN adheres to the principles set out in the following laws, regulations, and policies governing the care and use of laboratory animals: Italian Governing Law (D.lgs 26/2014; Authorization n.19/2008-A issued March 6, 2008 by Ministry of Health); Mario Negri Institutional Regulations and Policies providing internal authorization for persons conducting animal experiments (Quality Management System Certificate–UNI EN ISO 9001:2008 –Reg. N° 6121); the NIH Guide for the Care and Use of Laboratory Animals (2011 edition) and EU directives and guidelines (EEC Council Directive 2010/63/UE). The Statement of Compliance (Assurance) with the Public Health Service (PHS) Policy on Human Care and Use of Laboratory Animals has been recently reviewed (9/9/2014) and will expire on September 30, 2019 (Animal Welfare Assurance #A5023-01). The animals were regularly checked by a certified veterinarian responsible for health monitoring, animal welfare supervision, experimental protocols and procedure revision. All surgeries were performed under general anesthesia, and all efforts were made to minimize suffering.

### Study design

Forty 12-weeks-old male Sprague Dawley rats (*Rattus norvegicus*) (Envigo, Huntingdon, UK) (mean body weight 347 ± 9 g) were used in this study. In the absence of suitable data to perform the power analysis, the sample size was calculated according to the Mead’s resource equation (*E = N-T*, 10<*E*<20). Thus, considering two treated limbs per animal for a total of 16 treated tendons per time-point, the sample size was calculated as follows: *E* = (16–1)-(4–1) = 12. The rats were randomly divided into two treated groups that were injected within the Achilles tendon with collagenase type I (collagenase from *Clostridium histolyticum*, Worthington Lakewood, NJ, USA, 185 IU/mg): 1) collagenase 1 mg/mL defined as low dose (LD); 2) collagenase 3 mg/mL defined as high dose (HD). In each group, the contralateral tendon was treated either with phosphate buffered saline (PBS) and served as control (CTRL), or left untreated and served the sham control (S-CTRL). For each time point, 4 tendons were injected with LD, 4 tendons with HD, 4 tendons received PBS and 4 tendons were left untreated (n = 4). Tendon explants were analyzed at 3, 7, 15, 30 and 45 days.

### *In vivo* procedures

**Fig 1:**
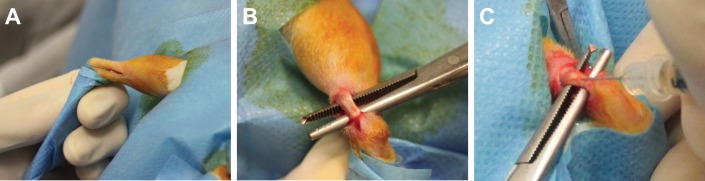
Surgical approach. A) Longitudinal incision of the skin in the medial portion of the hind limb. B) Achilles tendon exposure by blunt dissection. C) Injection of collagenase type I within the Achilles tendon structure.

The rats were anesthetized via inhalation of isoflurane (3%; Merial, Duluth, Georgia, USA) and maintained with an intraperitoneal injection of ketamine hydrochloride (80mg/kg; Imalgene, Merial, Milan, Italy) and medetomidine hydrochloride (1mg/kg; Domitor, Pfizer, New York City, NY, USA). All animals also received a preoperative intramuscular single injection of cefazolin (30mg/kg; Cefamezin, Teva, Petah Tikva, Israel). Using aseptic technique, after shaving and disinfection, a longitudinal 0.5 cm skin incision was performed through a medial approach to expose by blunt dissection the central region of the Achilles tendon. Then, all animals were injected with LD or HD of type I collagenase dissolved in 30 μL of PBS by means of a 30G needle into the central portion of the tendon, and with PBS into the contralateral tendon as controls (Fig 1). Finally, the skin was sutured by separated stitches with Prolene 4/0 (Johnson & Johnson, New Brunswick, NJ, USA). Atipamezole (1mg/kg; Antisedan, Pfizer, New York City, NY, USA) was administered subcutaneously to recover the animals from general anesthesia. After 3, 7, 15, 30 and 45 days, the rats were euthanized by CO_2_ inhalation to harvest the Achilles tendons for the histological investigations.

### Histological analysis

Tendon specimens were fixed in 10% formalin for 24h. Then, they were dehydrated in alcohol scale before embedding in paraffin and cutting into 5 μm longitudinal sections. The slides were stained with haematoxylin and eosin (H&E) to evaluate the tendon morphology of collagenase-treated groups compared to tendons treated with PBS (CTRL) or untreated native tendons (S-CTRL). Photomicrographs of the tissue were captured through an Olympus IX71 light microscope and an Olympus XC10 camera (Japan). Four sections of each sample were randomly selected and evaluated by two blinded observers to assess the tendon morphology according to a modified semi-quantitative grading score from 0 to 3 proposed by others [21, 22] (see S1 Table). The score analyzed the fiber structure and arrangement, resident cell density and appearance, infiltration of inflammatory cells, neovascularization and fatty deposits. According to this grading system, a perfectly normal tendon obtained score 0, whereas a score of 18 was assigned to maximally abnormal tendons.

### Statistical analysis

Comparisons between groups and time points were analyzed by the Mann-Whitney U test (GraphPad Prism v5.00 Software, La Jolla, CA, USA). All data are expressed as means ± standard deviation (SD). Values of p<0.05 were considered statistically significant. The interrater reliability of the examiners’ scores for each technique was calculated with intraclass correlation coefficient (ICC): ICC = 1, perfect reliability; ICC > 0.75, excellent reliability [23].

## Results

### Histopathological findings

**Fig 2:**
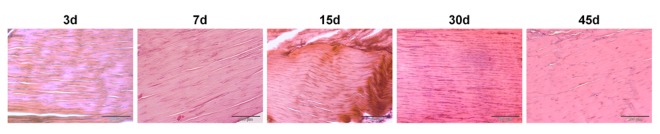
Histologic appearance of S-CTRL tendons at different time points. Representative micrographs of the histopathological analysis; H&E staining. Scale bars 200 μm (10X).

The S-CTRL tendons showed a uniform appearance of compact, well-aligned collagen fibers with a normal presence of spindle-shaped tenocytes disposed parallel to the fiber pattern, and, as expected, no degenerative events were observed during the whole study period (Fig 2).

**Fig 3:**
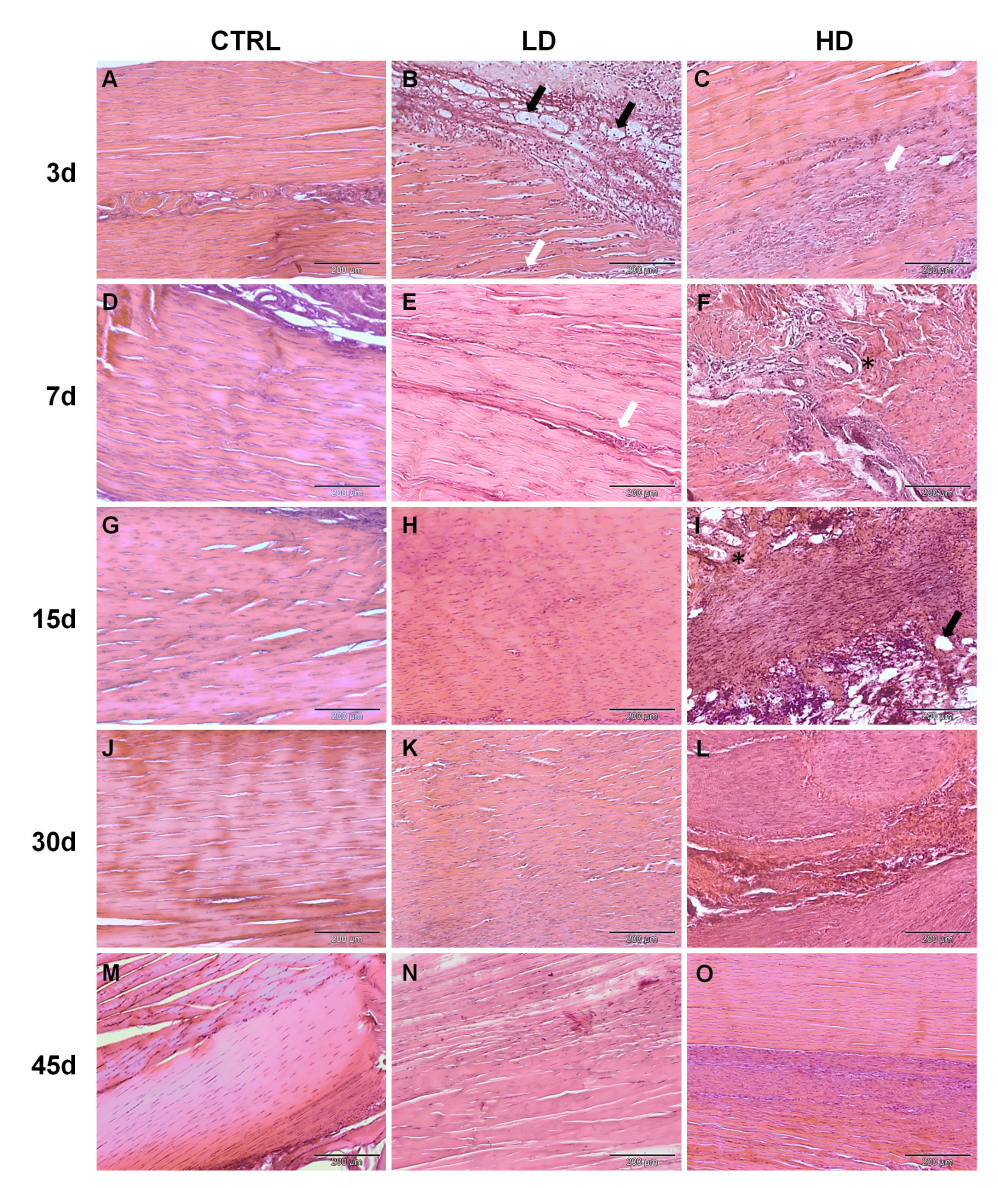
Histological appearance of CTRL, LD and HD-treated tendons at different time points. Representative micrographs of the histopathological analysis, H&E staining. Black arrows indicate fatty deposits; White Arrows: representative zone with high cellularity; *: vessels. Scale bars 200 μm (10X).

Differently, at day 3, the CTRL group exhibited a loss of fiber organization that also appeared partially fragmented along the route of the needle injection (Fig 3A). However, a complete recovery of the tendon structure was spontaneously achieved starting from day 7 (Fig 3D) and maintained until the last time point (day 45; Fig 3A).

The LD-treated tendons showed a mild degeneration throughout the time points. At day 3, the LD-treated tendons showed an abnormal presence of fatty deposits associated with the loss of fiber organization (Fig 3B, black arrows) disseminated with an increased number of slightly rounded resident cells (Fig 3B, white arrow). At later time points, the presence of a high number of rounded cells was the most distinctive feature of degeneration in these samples, by the way decreasing progressively until day 45 (Fig 3E, 3H, 3K and 3N).

Three days after the collagenase injection, the HD-treated tendons exhibited a moderate to marked collagen matrix disorganization with a great increase of cell density consisting mostly in rounded cells (Fig 3C). Moreover, a marked neoangiogenesis associated with the presence of several inflammatory cells was found (Fig 3C, white arrow). At days 7 and 15, the HD-treated tendons showed a complete fiber disorganization, in which the pattern was no more identifiable and the increased resident cells showed a rounded morphology (Fig 3F and 3I). Particularly on day 7, a marked increase in vascularity was detected related to the presence of several inflammatory cells (Fig 3F, asterisk). Furthermore, at day 15, a substantial presence of lipid vacuoles was found (Fig 3I, black arrow). Thirty days after the HD collagenase injection, the collagen fibers appeared disseminated of rounded and proliferative cells towards a new connective tissue deposed within the disrupted collagen fibers (Fig 3L). From day 30 to day 45, the collagen structure was resized and reshaped in a parallel organization, and a decreased cell number was detected at 45 days (Fig 3O).

In general, comparing the two treated groups, the HD collagenase induced a progressive degeneration of the tendon tissue, with a peak around day 15, while the injection of LD collagenase exerted its effect earlier (day 3) with no further increase of the pathological aspects during the following time points. Moreover, the LD treatment produced a mild degeneration with respect to the HD, particularly in terms of angiogenesis, fatty infiltration and fiber disorganization. Indeed, even if both treatments led to a spontaneous regeneration of the tissue at day 45, the effect of the treatment was still evident in the HD group, while the LD-treated tendons appeared similar to the CTRL (Fig 3N and 3O).

### Histopathological score analysis

**Fig 4:**
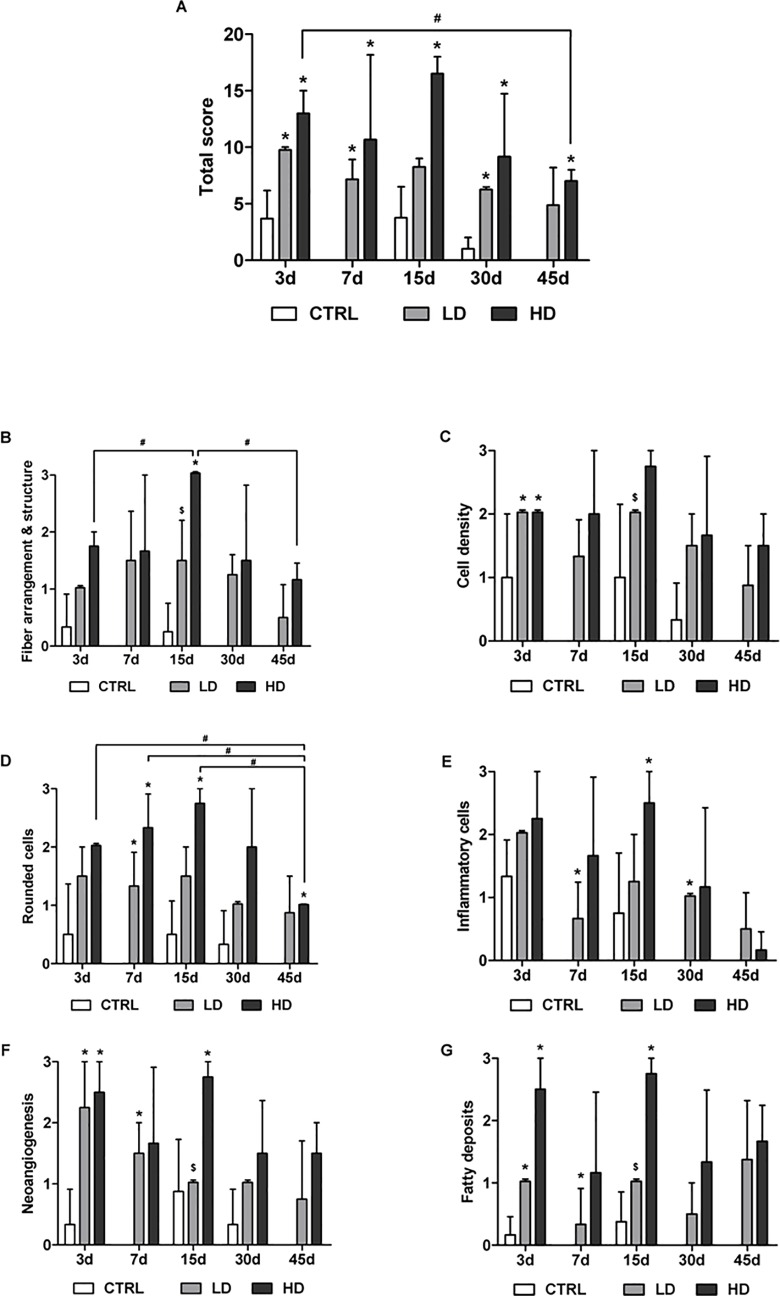
Histological scores. Total (A) and specific scores for fiber arrangement (B), cell density (C) and morphology (D), presence of inflammatory cells (E), neoangiogenesis (F) and fatty deposition (G). Data are reported as mean±SD. *p<0.05 respect CTRL; ^$^p<0.05respect to HD; ^#^p<0.05 respect to different time point; n = 4.

The interrater reliability of the scoring analysis performed by two blinded examiners was good (ICC 0.73). The total and specific histopathological scores are presented in the histograms in Fig 4A.

The injection of collagenase induced deep changes in the histological appearance of the treated tendons resulting in a significantly worse total score of the HD and LD groups compared to the CTRL group at all time-points (with the exception of LD at day 15; Fig 4A). The HD group reached the worst total score at day 15 (16.5±2.1), while the LD one reached a maximum of 9.7±0.4 at day 3. Remarkably, the total score in the HD group decrease significantly between day 3 and 45 (p<0.05).

Both concentrations of collagenase produced a visible damage on fiber arrangement, though significant differences were just observed at day 15 in the HD group respect to CTRL (p<0.05) and to LD (p<0.05). Analyzing the temporal changes in terms of disorganization of collagen fibers, we found a progressive score worsening at least up to 15 days (1.75±0.4 at day 3, 3±0.0 at day 15, p<0.05), but then a subsequent spontaneous regeneration was observed (1.17±0.3 at day 45, p<0.05) (Fig 4B).

At day 3, cell density was increased by all the treatments with a significant increase between CTRL and HD (p<0.05) or LD groups (p<0.05) (Fig 4C). The cell density of the HD group was higher at all time-points respect to CTRL and LD treatments, with a significant increase respect to LD at day 15 (p<0.05).

Cell morphology was also affected by the treatments. In fact, a higher number of rounded cells was observed in the LD and HD groups with respect to CTRL at all time-points (Fig 4D). In particular, at day 7, both LD and HD group showed a higher score with respect to CTRL (p<0.05). Moreover, the HD group showed significant increases also at day 15 and 45 compared to CTRL (p<0.05). Nevertheless, in the HD group, a progressive decrease of rounded cells was observed at day 45 with respect to day 3, 7 and 15 (p<0.05).

The infiltration of inflammatory cells was observed in all samples at day 3. By day 7, they almost disappeared in CTRL group, while they were persistent in LD and HD ones. In particular, in the LD group, a significant increase was observed with respect to CTRL at day 7 and day 30 (p<0.05), whereas in the HD group it was found only at day 15 (p<0.05) (Fig 4E).

The presence of new vessels was evident in all collagenase-treated tendons (Fig 4F), specifically, their number significantly increased in both LD and HD groups with respect to CTRL up to 15 days (p<0.05). In particular, HD groups showed a greater presence of vessels compared to LD group at day 15 (p<0.05). A similar behavior was observed in terms of fatty deposits (Fig 4G). In fact, at day 3, both doses of collagenase caused greater fatty deposits with respect to CTRL (p<0.05). Their increase was found at day 7 in the LD group and at day 15 in the HD group with respect to CTRL. The HD group showed higher levels of fatty deposits at all time-points, in particular, a significant difference was observed with respect to LD at day 15 (p<0.05).

Overall, the LD-treated tendons showed a lower degenerative score with respect to the HD group. Moreover, the pathological changes in tendons treated with LD collagenase did not significantly vary during the experimental observation with any differences observed between time points neither for the total score nor for the specific features.

## Discussion

Tendinopathy is an umbrella term that refers to tendon injury with unknown etiology [24]. More precisely, tendinopathy has been defined as tendinitis when a non-rupture tendon injury is associated with a very precocious inflammatory process [25]. This process brings to mechanical-related chronic lesions, commonly known as tendinosis [25]. The lack of knowledge about the physiopathology of tendinopathy leads to misleading opinions in the presence of a host inflammatory response and development phases of this disorder. In this context, the current failure to offer a complete clinical picture of such a multiple etiology disease [14, 26] increases the need of a valid animal model able to mimic the histological features of tendinopathy in humans and to establish a standardized tool suitable for future preclinical studies.

Collagenase is considered an effective method to induce the Achilles tendinopathy in preclinical models, as widely described in the literature [14, 15, 17–21, 27–30]. However, the use of different rodent strains, different protocols in terms of amount and type of collagenase administration and follow-up time points determined a poor reproducibility of the model [13].

Aiming at standardizing a rat model of collagenase-induced tendinopathy, we carefully compared the degenerative potential of two different concentrations of collagenase type I. In particular, we provided a complete time course evaluation of collagenase-induced Achilles tendinopathy in rats, focusing on the tendon histopathology at different time points (3, 7, 15, 30 and 45 days), in order to define the most effective dose of collagenase at an exact temporal window able to generate histological evidences of tendon lesions.

The choice of the collagenase doses included in this work was based on our previous *in vitro* studies, in which the dose inducing the collagen fiber disruption was validated in tendon explants [31, 32].

Our results revealed that both LD- and HD-treated tendons displayed a disorganization of the collagen fibers and increased the number of rounded resident cells, suggesting that a single intra-tendinous injection was sufficient and effective to induce a prompt and severe impairment of Achilles tendon integrity, above all in tendons treated with HD of collagenase type I. Our findings were consistent with other studies performed in large animals, in which the severity of the pathology was related to the amount of the injected collagenase [33]. The morphological changes—especially occurring at day 15—resembled the histological appearance of tendinopathy in humans. In fact, human Achilles tendinopathy exhibits disorganized and smaller collagen fibers, loss of their parallel orientation, and an increased amount of rounded-shaped tenocytes [6].

Overall, in this model, we demonstrated that the acute phase occurred from day 3 to day 15 and evolved towards a proliferative phase from day 15 to day 45 displaying a degenerative appearance associated to a very precocious and mild remodeling process, according to what observed by previous *in vivo* and human studies [6, 34]. Moreover, 25% of cases treated with both LD and HD collagenase showed the presence of chondrocyte-like cells disseminated within the damaged tendon fibers (see S1 Fig). This finding was consistent with other studies describing both the presence of chondrocytes and the up-regulation of chondrogenic genes in rat patellar and Achilles tendons after four weeks of collagenase injection [14, 27]. Similarly, chondrocyte markers were also expressed in human clinical samples of patellar [35], supraspinatus [36] and Achilles tendons [6]. The impairment of the tendon feature in favor of a fibrocartilaginous one assumes a pathological significance. Indded, the progressive lack of elasticity and tensile strength makes tendon more subjected to ruptures, even if no molecular mechanisms and pathways occurring in human Achilles tendinopathy have been investigated yet [37]. This condition was strengthened by some *in vitro* studies that demonstrated the capability of tendon stem/progenitor cells to effectively transdifferentiate towards the chondrogenic lineage [38–40].

The presence of fatty deposits was observed in tendons treated with HD collagenase, while they were rarely found in the LD-treated ones. The fat infiltration is retrieved in case of poor tendon repair. Moreover, fatty deposits were found in chronic tendinopathy in humans and in large animals [35, 41].

Our findings showed increased neo-angiogenesis in collagenase treated samples, more markedly visible in the HD-treated tendons than in the LD ones, above all at the earlier stages of the disease. The presence of new vessels was mainly restricted to the peritenon and it was combined with an initial increase of the cell number from day 3 to day 15, followed by a decrease from day 15 to day 45. The neovascularization and the increased amount of cells could be correlated to an inflammatory reaction. Our data were supported by several studies that demonstrated an inflammatory reaction in humans and animal models, both in the early overload response and in the established tendinopathy [42]. However, the role of inflammation in tendon healing is still a greatly debated topic. Inflammation is highly beneficial to the tissue repair thanks to the release of cytokines and growth factors that together promote neoangiogenesis and the recruitment of resident and progenitor cells, and macrophages [43]. Nevertheless, how the inflammatory reaction can influence the progression of the pathology and how it can possibly contribute to the healing process are still unanswered questions. So, since its role in tendinopathy is uncertain, the presence (or absence) of an inflammatory response in our model would not have represented a crucial parameter to be considered in comparison with the human disease, thus, it was not deeply investigated. Despite our study was based only on histological evaluations, it was able to resemble the most important tendinopathy features in terms of tissue damages. These outcomes need to be examined in depth through quantitative analyses to assess the correlation of the histological findings with biochemical analyses, such as glycosaminoglycan, collagen and DNA contents, as well as with biomechanical parameters.

Overall, the results of this study suggested that the HD collagenase-induced tendinopathy is a reliable model in rats, resembling the human disease. In particular, our results demonstrated that collagenase type I efficiently induced three distinct stages of the disease over time, thus offering the opportunity to accurately investigate the pathological progression in a well-controlled establishment of this complex injury. More importantly, this model could be used to test novel therapies during the three-stage tendon disorder to achieve the most effective results in patients.

## Supporting Information

S1 Fig Chondrocyte-like cells in LD and HD treated tendon at day45. Representative micrographs of the histopathological analysis; H&E staining. Scale bars 100 μm (20X).
(TIF)

S1 Table Grading system for the tendon histological evaluation. (DOCX)

## Abbreviations

LD: Low dose
HD: high dose
PBS: phosphate buffered saline
CTRL: control
S-CTRL: sham control
H&E: haemoatoxylin and eosin
SE: standard error
ICC: interclass correlation coefficient
