# Supplementary figures and images for: Dose-Related and Time-Dependent Development of Collagenase-Induced Tendinopathy in Rats
Source: PLoS One. 2016 Aug 22;11(8):e0161590. doi: 10.1371/journal.pone.0161590 (PMC4993508; doi:10.1371/journal.pone.0161590)

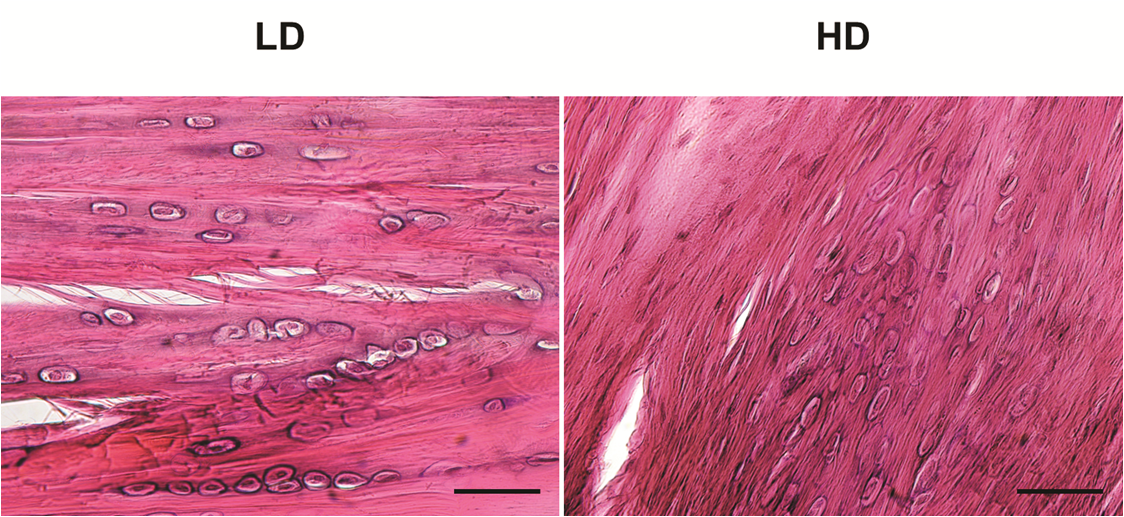

Supplement: S1 Fig — Representative micrographs of the histopathological analysis; H&E staining. Scale bars 100 μm (20X). (TIF) [file pone.0161590.s001.tif]
