# Supplementary material for: Dose-Related and Time-Dependent Development of Collagenase-Induced Tendinopathy in Rats
Source: PLoS One. 2016 Aug 22;11(8):e0161590. doi: 10.1371/journal.pone.0161590 (PMC4993508; doi:10.1371/journal.pone.0161590)
Supplement: S1 Table — (DOCX) [file pone.0161590.s002.docx]

**S1 Table. Grading system for the tendon histological evaluation**

|  | **0** | **1** | **2** | **3** |
| --- | --- | --- | --- | --- |
| **Fiber structure and arrangement** | Normal: continuous, parallel collagen fibers | Slightly abnormal: partially disorganized and fragmented fibers | Abnormal: moderately disorganized, fragmented, crossed and wavy fibers | Markedly abnormal: total disorganized and non-identifiable fiber pattern |
| **Cell density** | Normal | Slightly increased | Moderately increased | Markedly increased |
| **Cell appearance** | Spindle-shape cells | Slightly rounded cells | Moderately rounded cells | Markedly rounded cells |
| **Inflammatory cell infiltration** | <10% | 10-20% | 20-30% | >30% |
| **Neovascularization** | Normal presence of vascular bundles | Slight increase of vascular bundles | Moderate increase of vascular bundles | Marked increase of vascular bundles |
| **Fatty deposits** | Absence of lipid vacuoles | Slight increase of lipid vacuoles | Moderate increase of lipid vacuoles | Marked increase of lipid vacuoles |
